# Disorders of sodium balance in COVID-19 patients: two Tunisian patients report

**DOI:** 10.11604/pamj.2021.39.199.27626

**Published:** 2021-07-14

**Authors:** Ghada Saad, Asma Ben Abdelkrim, Yosra Hasni El Abed, Soumaya Tahri, Asma Gorchene, Amel Maaroufi, Molka Chadli, Maha Kacem Njah, Koussay Ach

**Affiliations:** 1 Endocrinology and Diabetology Department, Farhat Hached Hospital of Sousse, Sousse, Tunisia

**Keywords:** Hyponatremie, hypernatremie, SARS-CoV-2, COVID-19, Tunisia

## Abstract

Coronavirus disease 2019 (COVID-19) was first reported in December 2019. The disease is caused by severe acute respiratory syndrome virus corona virus 2 (SARS-CoV-2). Mild respiratory symptoms are the most common manifestations of SARS-CoV-2, but new signs are constantly being discovered as it spreads. Disorders of sodium balance are increasingly described in patients with SARS-CoV-2. We report, here, the cases of two patients presented with COVID-19 and in whom we discovered sodium disorders. The first patient is a 74-year-old man who presented with fatal hypernatremia. The second patient is a 66-years-old man presented with COVID-19 and euvolemic hyponatremia attributed to syndrome of inappropriate anti-diuretic hormone secretion (SIADH). This hyponatremia persisted long after the respiratory signs disappeared. Sodium balance disorders are increasingly described in the literature; special attention should be paid to the electrolyte status of COVID-19 patients. Pathophysiological mechanisms associating SARS-CoV-2 with these disorders are being studied.

## Introduction

The global spread of severe acute respiratory syndrome coronavirus 2 (SARS-CoV-2), its severity and high mortality rate have been subject of much writing. It is a potentially serious disease that can affect many tissues and systems. Several mechanisms have been implicated. SARS-CoV-2 infects human cells by binding to angiotensin-I converting enzyme 2 (ACE2), the principal axis of the renin-angiotensin system (RAS) which is an essential factor in controlling blood pressure and electrolyte balance [1]. Mild respiratory symptoms are the most common manifestations of SARS-CoV-2, but new signs are constantly being discovered as it spreads. Sodium balance disorders have not been widely described. Sodium is an important cation that is distributed primarily outside the cell. Aldosterone acts on the sweat ducts and colonic epithelium to conserve sodium. When aldosterone is activated to retain sodium the plasma sodium tends to rise. This immediately causes the release of ADH, which causes water to be retained, thus balancing Na+ and H2_O_in the right proportion to restore plasma volume. We describe two patients with corona virus disease 2019 (COVID-19) who presented dysnatremia: hypernatremia in the first patient and hyponatremia in the second.

## Methods

During the period between September and December 2020, we took care of 87 patients with COVID-19 in our department. Among these patients, we found 2 who presented with dysnatremia: hypernatremia in the first and hyponatremia in the second. We report them to the relevance of these sodium disorders in the context of the SARS-CoV-2 pandemic.

## Results

**Case 1:** our first patient is a 74-year-old Tunisian man known to have type 2 diabetes and hypertension. He was presented with an 8-day history of progressive asthenia, myalgia, headache and fever due to SARS-CoV-2 infection. On the day of admission, he had developed dyspnea. The chest computed tomography (CT) showed involvement of 75% of lung parenchyma. The patient was put on oxygen because he was polypneic at 30 cycles per minute with oxygen saturation of 70% on room air. He was treated with 16 liters per minute of oxygen on non-rebreather, cefotaxime 3 g/day, dexamethason 8 mg/day and enoxaparin 6000 IU twice a day. Initial laboratory investigations showed mild hyponatremia of 134 mmol/l (135 - 145 mmol/l). On the 3^rd^ day of hospitalization, the patient presented, suddenly, mental confusion and agitation with normal hemodynamic status: BP = 140/90 mmHg HR = 89 bpm Laboratory investigations were remarkable for severe hypernatremia >150 mmol/l (135 - 145 mmol/l); glycemia = 9 mmol/l; kalemia = 5 mmol/l (3.5 - 5 mmol/l); calcium = 2.78 mmol/l (2.2 - 2.6 mmol/l); creatinine = 197 µmol/l; urea = 31.6 mmol/l; D-dimer = 2276 ng/ml; plasmatic osmolarity > 349 mosmol/l; ABG: pH = 7.35 PCO_2_= 31mmHg PaO_2_= 83mmHg. The patient died before the laboratory results arrived.

**Case 2:** the second patient is a 66-years-old Tunisian man with COVID-19 known to have type 2 diabete on glimepiride, hypertension on converting enzyme inhibitor (CEI), controlled hypothyroidism and coronary insufficiency on clopidogrel. He was presented with a 2 days history of progressive asthenia, digestive symptoms and fever. On the day of admission, he had developed dyspnea. At the initial examination, the patient was febrile, polypneic, with blood pressure of 110/80 mmHg, heart rate of 88 beats per minute and respiratory rate of 22 breaths per minute with oxygen saturation of 94% on room air. He did not have any signs of fluid overload or dehydration. He was treated with 6 liters per minute of oxygen on non-rebreather, insulin, cefotaxime, azithromycin, hydroxychloroquine, dexamethason, enoxaparin, CEI, levothyroxin and clopidogrel. Initial laboratory investigations showedhyponatremia of 126 mmol/l; glycemia = 10.9 mmol/l; kalemia = 3.6 mmol/l; calcemia = 2.2 mmol/l; creatinin = 79 µmol/l; urea =3.7 mmol/l; plasmatic osmolarity = 273 mosmol/l; natriuresis = 48 mmol/d; urinary osmolarity = 452 mosmol/l; TSH = 3.6 mUI/l; pH = 7.4; PCO_2_= 33 mmHg. These biological abnormalities correspond to a SIADH. The treatment was based on fluid restriction and maintaining osmolar intake (sodium intake) normal. Hyponatremia persisted throughout follow-up and resolved spontaneously after 2 months.

## Discussion

**Figure 1:**
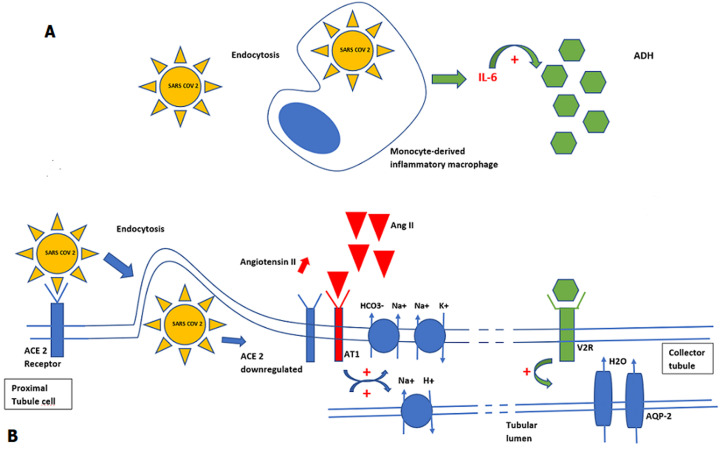
simplified potential role of SARS-CoV-2 in the genesis of sodium disorders, A) potential mechanism of hypernatremia; B) potential mechanism of hyponatremia. ACE 2: angiotensin-converting enzyme 2; Ang II: angiotensin II; AT1: angiotensin II receptor type 1; ADH: anti-diuretic hormone; V2R: vasopressin 2 receptor; AQP-2: aquaporin 2

The most common clinical characteristics of COVID-19 are fever, dry cough, myalgia, anorexia and dyspnea and gastrointestinal symptoms [2]. Many other new signs are discovered every day. Sodium disorders have recently started to draw attention to their relationship with SARS-CoV-2. We report the cases of two patients who were treated in our department for COVID-19 and in whom we discovered sodium disorders, hypernatremia in the first case and hyponatremia in the second one. Hypernatremia was observed on patients with severe COVID-19. This entity was reported for the first time by Milena *et al*. [3] then by Weihua *et al*. [4]. The incidence of hypernatremie in patient with COVID-19 was higher than the reported one in medical ICU setting 50% vs 26% [3,4]. Hypernatremia in medical intensive care settings is often iatrogenic; which is not the case with our patient. Elevated plasma creatinine concentration in our patient reflects renal injury. It is known that SARS-CoV-2 binds to the angiotensin-converting enzyme 2 (ACE2), receptor which is highly expressed in the kidneys, specifically in the proximal tubule [5,6]. Identification of SAR-CoV-2 ribonucleic acid (RNA) in the urine of an infected patient shows that the virus can enter the tubular fluid where it may bind to those ACE2 receptors in the proximal tubule [7]. Following endocytosis of the viral complex, ACE2 is down regulated resulting in angiotensin II accumulation [8]. Angiotensin II facilitates sodium reabsorption by stimulating sodium-hydrogen exchange in the proximal convoluted tubule of the kidney [8] (Figure 1A).

Our patient did not have saline infusion, and the received drugs do not give hypernatremia. Therefore, we do not find a physiopathological explanation apart from the involvement of SARS-CoV-2 in sodium regulation. The reported cases with hypernatremia and COVID-19 had all severe pulmonary involvement and were managed in medical ICU. Ruiz-Sanchez *et al*. [9] had shown in their study that there was an increased mortality in hypernatremic patients as compared with eunatremic patients, hypernatremia was an independent risk factor for a higher mortality rate. Severe hypernatremia is life-threatening, the death of our patient was the proof. Further studies including patients with hypernatremia and COVID-19 are needed to establish this relationship firmly. In addition to hypernatremia, hyponatremia is the second sodium abnormality that we describe in this paper. Data from the USA showed that hyponatremia occurred in 50% of patients admitted with COVID-19 [10]. It is shown to be more frequent then hypernatremia in published studies [4,9]. Euvolemic hyponatremia was the most common dysnatremia in patients with COVID-19 treated by Weihua *et al*. [4] and Ruiz-Sánchez *et al*. [9].

Many case series of COVID-19 pneumonia associated with syndrome of inappropriate anti-diuretic hormone secretion (SIADH) was published [11]. Habib *et al*. even reported a case of infection with SARS-CoV-2 revealed by hyponatremia due to SIADH [12]. Hyponatremia in SIADH patients can be caused by several etiologies such as malignancy, pulmonary conditions, central nervous system disorders, and medications [13]. Viral pneumonia is one of the common causes for SIADH. In our patient, vomiting and diarrhea could explain the hyponatremia. However, the low serum osmolarity coupled within appropriate urinary osmolarity in the setting of severe hyponatremia was suggestive of SIADH. Our patient was diagnosed as SIADH based on hyponatremia (serum sodium level <135 mmol/l), serum osmolality <275 mOsm/kg, urine osmolality >100 mOsm/kg, urine sodium concentration >40 mmol/l, normokalemia and euvolemic state with normal renal, adrenal, and thyroid functions. All medications our patient was taking are not implicated to induce hyponatremia.

The hypothesized mechanisms for SIADH in COVID-19 infection include inflammatory cytokine release, ventilation-perfusion mismatch, intravascular volume depletion etc. [11,14]. In inflammatory conditions, interleukin-6 (IL-6) released by monocytes and macrophages, plays a pathogenic role in causing electrolyte impairment by inducing the non-osmotic release of vasopressin [15] (Figure 1B). We know thatIL-6 is one of the most important cytokines involved in COVID-19-induced pathology [16]. Berni *et al*. [16] observed the course of hyponatremia in patients treated with tocilizumab and concluded that the significant increase of natremia after 48 h from the initiation of tocilizumab treatment suggests the presence of an association betweenIL-6, vasopressin release and natremia. Inflammatory cytokine release, in particular IL-6, is therefore the most probable hypothesis in the genesis of euvolemic hyponatremia in patients infected with SARS-CoV-2. Hyponatremia, whatever its cause, has been clearly associated with severe forms and mortality from COVID-19 regardless of all other serious risk factors [9].

In the study conducted by Ruiz-Sánchez *et al*. [9] which included 4,664 patients, analysis indicated that chronic kidney disease and bilateral pneumonia, as well as tachypnea, male sex, and an age ≥70 years, were linked to decreased plasma sodium levels or the presence of hyponatremia. A correlation between the degree of involvement of the pulmonary parenchyma and the level of natremia has not been studied. Based on these observations we can recommend an analysis of the blood ionogram at the hospitalization of patients with COVID-19. The presence of hyponatremia or hypernatremia is predictive of a worsening of the patient's condition. Special attention should be paid to elderly patients, those with severe lung disease and those with risk factors for developing dysnatremia. It has been observed that the correction of sodium disorders in patients in intensive care settings improves the survival rate [17]; this should be tried in patients with COVID-19.

## Conclusion

During the ongoing COVID-19 pandemic, disorders of sodium balance could be unusual presenting features of SARS-CoV-2 infection. Raising awareness about this association may lead to faster diagnosis and management. The involvement of SARS-CoV-2 in these sodium balance disorders needs to be further investigated. A large-scale analysis of patients hospitalized for COVID-19 may answer several questions and identify if sodium correction impacts outcome.

### What is known about this topic

- There are many clinical and biological manifestations of SARS-CoV-2, and some are still being discovered;
- Sodium disturbances have been observed in patients with COVID-19 and have been linked to severe forms.

### What this study adds

- Thanks to our case reports, we can offer recommendations concerning the need to prescribe an ionogram in some patients with COVID-19;
- We also propose a simplified figure illustrating the mechanisms leading to dysnatremia in patients with COVID-19.

## References

[1] Chen L, Li X, Chen M, Feng Y, Xiong C (2020). The ACE2 expression in human heart indicates new potential mechanism of heart injury among patients infected with SARS-CoV-2. Cardiovasc Res.

[2] Wang D, Hu B, Hu C, Zhu F, Liu X, Zhang J (2020). Clinical characteristics of 138 hospitalized patients with 2019 novel coronavirus-infected pneumonia in Wuhan, China. JAMA.

[3] Zimmer MA, Zink AK, Weißer CW, Vogt U, Michelsen A, Priebe HJ (2020). Hypernatremia-a manifestation of COVID-19: a case series. A&A Pract.

[4] Hu W, lv X, Li C, Xu Y, Qi Y, Zhang Z (2021). Disorders of sodium balance and its clinical implications in COVID-19 patients: a multicenter retrospective study. Intern Emerg Med.

[5] Zhang H, Penninger JM, Li Y, Zhong N, Slutsky AS (2020). Angiotensin-converting enzyme 2 (ACE2) as a SARS-CoV-2 receptor: molecular mechanisms and potential therapeutic target. Intensive Care Med.

[6] Lan J, Ge J, Yu J, Shan S, Zhou H, Fan S (2020). Structure of the SARS-CoV-2 spike receptor-binding domain bound to the ACE2 receptor. Nature.

[7] Peng L, Liu J, Xu W, Luo Q, Chen D, Lei Z (2020). SARS-CoV-2 can be detected in urine, blood, anal swabs, and oropharyngeal swabs specimens. J Med Virol.

[8] Kai H, Kai M (2020). Interactions of coronaviruses with ACE2, angiotensin II, and RAS inhibitors-lessons from available evidence and insights into COVID-19. Hypertens Res.

[9] Ruiz-Sánchez JG, Núñez-Gil IJ, Cuesta M, Rubio MA, Maroun-Eid C, Arroyo-Espliguero R (2020). Prognostic impact of hyponatremia and hypernatremia in COVID-19 pneumonia. A hope-COVID-19 (Health Outcome Predictive Evaluation for COVID-19) Registry Analysis. Front Endocrinol (Lausanne).

[10] Aggarwal S, Garcia-Telles N, Aggarwal G, Lavie C, Lippi G, Henry BM (2020). Clinical features, laboratory characteristics, and outcomes of patients hospitalized with coronavirus disease 2019 (COVID-19): early report from the United States. Diagnosis(Berl).

[11] Yousaf Z, Al-Shokri SD, Al-Soub H, Mohamed MFH (2020). COVID-19-associated SIADH: a clue in the times of pandemic!. Am J Physiol Endocrinol Metab.

[12] Habib MB, Sardar S, Sajid J (2020). Acute symptomatic hyponatremia in setting of SIADH as an isolated presentation of COVID-19. IDCases.

[13] Decaux G, Musch W (2008). Clinical laboratory evaluation of the syndrome of inappropriate secretion of antidiuretic hormone. Clin J Am Soc Nephrol.

[14] Khalangot M (2020). COVID-19 and SIADH relations: impact of the positive pressure ventilation. Am J Physiol Endocrinol Metab.

[15] Hodax JK, Bialo SR, Yalcindag A (2018). SIADH in systemic JIA resolving after treatment with an IL-6 inhibitor. Pediatrics.

[16] Berni A, Malandrino D, Parenti G, Maggi M, Poggesi L, Peri A (2020). Hyponatremia, IL-6, and SARS-CoV-2 (COVID-19) infection: may all fit together?. J Endocrinol Invest.

[17] Hoorn EJ, Lindemans J, Zietse R (2006). Development of severe hyponatraemia in hospitalized patients: treatment-related risk factors and inadequate management. Nephrol Dial Transplant.

